# Adiponectin receptor agonist reduces broiler hepatic lipid deposition

**DOI:** 10.3389/fvets.2025.1667501

**Published:** 2025-09-19

**Authors:** Wenhao Tan, Kunyu Jiang, Yuhan Zhang, Hang Gao, Xingyi Tang, Sha Jiang

**Affiliations:** ^1^Joint International Research Laboratory of Animal Health and Animal Food Safety, College of Veterinary Medicine, Southwest University, Chongqing, China; ^2^Immunology Research Center, Medical Research Institute, Southwest University, Chongqing, China

**Keywords:** adiponectin receptor agonist, broiler, chicken hepatocellular carcinoma cell, fatty liver syndrome, corticosterone

## Abstract

To investigate the effects of AdipoRon on fatty liver syndrome (FLS) in chicken, we used a corticosterone (CORT)-induced fatty liver model in Cobb broilers *in vivo* and fat emulsion-induced model in Leghorn male hepatoma cells (LMH) *in vitro*. In the *in vivo* study, eighteen 33-day-old male Cobb broilers were randomly assigned to three groups: control group (CONT, vehicle), corticosterone-treated group (CORT, 4 mg/kg), and corticosterone with AdipoRon-treated group (CORT-AR, 4 mg/kg and 0.2 mg/kg, 1 time/1 day) for 5 days. The results showed AdipoRon reduced CORT-induced increase in liver crude fat content (*p* < 0.05), increased protein expressions of peroxisome proliferator-activated receptor *α* (PPARα) (*p* < 0.05) and adiponectin (ADPN) (*p* < 0.05), and suppressed the protein expressions of Acetyl-CoA carboxylase 1 (ACC) (*p* < 0.05) and phosphorylated c-Jun N-terminal kinase 1 (p-JNK1) (*p* < 0.05) in the liver. In the *in vitro* study, LMH cells were divided into control (CN), fat emulsion (FE, 10%), and FE + AdipoRon (4 μM) group (FE-AR). AdipoRon reduced FE-induced lipid accumulation (*p* < 0.05), decreased the protein expression of ACC and tumor necrosis factor-alpha (TNF-*α*), and enhanced PPARα, the phosphorylation of adenosine 5′-monophosphate-activated protein kinase (AMPK), and carnitine palmitoyl transferase 1 (CPT-1) (*p* < 0.05). In conclusion, AdipoRon effectively reduces hepatic lipid deposition in CORT-induced FLS broilers, likely through PPARα activation and inhibition of lipid synthesis via ACC downregulation.

## 1 Introduction

Fatty liver syndrome (FLS) is a common metabolic disorder in poultry characterized by disrupted lipid metabolism and excessive hepatic lipid accumulation, leading to impaired liver function, reduced productivity, meat quality, and increased susceptibility to disease (1–3). In a study of 76 deceased layer hens diagnosed with fatty liver hemorrhagic syndrome (FLHS) at a northern California facility, 69.7% of the birds were in the active laying period, indicating that FLHS significantly reduced egg production rates of the hens and negatively affected breeding efficiency (4). In chicken, FLS is often induced by nutritional imbalances, stress, or hormonal dysregulation (5). Addressing lipid accumulation and inflammatory reactions in the early stages of chicken liver is crucial for the prevention and treatment of FLS (6, 7).

Adiponectin (ADPN), an adipokine predominantly secreted by adipose tissue, plays a key role in maintaining lipid and glucose homeostasis (8). As a key metabolic organ, adipose tissue integrates signals from immune cells, neural inputs, and gene regulatory networks to maintain systemic energy balance (9–11). ADPN is an important mediator within this network, and its dysregulation, often associated with excessive lipid accumulation, can contribute to inflammation, insulin resistance, and metabolic disease (11, 12). ADPN exerts its effect by activating adenosine 5′-monophosphate-activated protein kinase (AMPK) and peroxisome proliferator-activated receptor *α* (PPARα) signaling pathways (13). In the ADPN-AMPK pathway, ADPN activates AMPK which triggers phosphorylation of Acetyl-CoA carboxylase 1 (ACC), increasing the production of carnitine palmitoyl transferase 1 (CPT-1), an enzyme essential for mitochondrial fatty acid uptake (14), ultimately promoting fatty acid oxidation and decreasing lipid synthesis in the liver (15). On the other hand, ADPN-PPARα signaling pathway also regulates hepatic fatty acid uptake and oxidation through acetyl CoA oxidase (ACO) and several other PPAR*α* targeted genes (16). Additionally, ADPN has been shown to reduce the plasma level of tumor necrosis factor-alpha (TNF-α) and counteract its damaging effects within the liver tissue (15, 16). Research suggested that ADPN may alleviate metabolic inflammation through suppression of c-Jun N-terminal kinase (JNK) signaling, a pro-inflammatory pathway involved in hepatic insulin resistance (17, 18).

Adiponectin receptor agonist (AdipoRon) is a synthetic small molecule compound that has been shown to mimic the effects of endogenous ADPN by activating the ADPN receptors R1 and R2 (AdipoR1/2) (19, 20). It has been reported that AdipoRon can downregulate the expression of fatty acid synthesis genes ACC and fatty acid synthetase (FAS), promote the phosphorylation of AMPK and ACC, and increase the protein level of PPARα, inhibiting lipid synthesis in the mouse hepatocyte line FL83B (21). Despite its promising potential in regulating hepatic lipid metabolism, research on the effects of AdipoRon in poultry remains limited. One study demonstrated positive effect of AdipoRon on reducing lipid content in the livers of geese fed with a high-fat diet (22). Another preliminary laboratory research showed that AdipoRon can reduce triglyceride (TG) content in primary chicken embryo liver cells induced by fat emulsion (23). However, its potential role in modulating FLS in broilers has not been well investigated.

Corticosterone (CORT) is the primary avian glucocorticoid (GC), playing a significant role in lipid metabolism disturbances (24, 25). Studies have shown that excessive CORT exposure promotes hepatic lipid accumulation by stimulating lipogenesis and suppressing fatty acid oxidation, ultimately leading to metabolic dysfunction in broilers. Consequently, CORT has been used to induce FLS in chickens (26, 27). In this study, we investigated the effects of AdipoRon on CORT-induced FLS in broilers and its underlying mechanisms using *in vivo* and *in vitro* experiments. It aimed to provide new insights into the potential application of adiponectin receptor agonists in poultry production, with implications for improving liver metabolic efficiency in broilers.

## 2 Materials and methods

### 2.1 Experimental animal management and design

All procedures in this experiment were approved by the Animal Ethics Committee of the Southwest University, Chongqing, China (the permission number: IACUC-20231023-05). The broilers used in this study had been raised together from hatch to 30 days of age under uniform husbandry conditions, including standardized feed, water, and environmental management, with routine vaccinations and veterinary health monitoring to ensure consistent health status. Eighteen 30-day-old broilers with similar weights were randomly divided into three treatment groups (*n* = 6 per treatment) for a total of 3 days of acclimation followed by 5 days of treatment. During the study period, the broilers were fed with a commercial broiler diet (Charoen Pokphand Feed Co., Ltd., Chongqing, China), with the composition detailed in Table 1. Following acclimation, the broilers received the following treatments: control group (CONT, vehicle solution, 1 time/day), corticosterone group (CORT, 4 mg/kg, 1 time/day, Aladdin, Shanghai, China), and corticosterone + AdipoRon group (CORT-AR, 4 mg/kg CORT and 0.2 mg/kg AdipoRon, 1 time/day, MedChem Express, Shanghai, China) (22). CORT was dissolved in 0.9% saline and administered via subcutaneous injection in the neck area. AdipoRon was dissolved in a vehicle solution composed of dimethyl sulfoxide (DMSO), polyethylene glycol 300 (PEG300), Tween-80, and 0.9% saline, and was administered via intraperitoneal injection. To mitigate procedural stress, broilers underwent habituation via sham injections prior to the study. Injection sites were systematically rotated to prevent localized tissue injury, followed by aseptic compression with sterile gauze. Animals were monitored throughout the entire experiment period for acute and chronic adverse effects.

**Table 1:** Feeding recipes.

Ingredient	511 compound feed
Water (%)	≤14
Crude protein (%)	≥19
Calcium (%)	0.6 ~ 1.2
Total phosphorus (%)	≥0.4
NaCl (%)	0.2 ~ 0.8
Crude fiber (%)	≤6.0
Coarse ash (%)	8.0
Methionine + cystine (%)	≥0.74

### 2.2 Experimental animal sample collection

Body weight (BW) of the broilers was measured and recorded on days 33 and 38. On day 38, the broilers were anesthetized with tiletamine-zolazepam (Zoletil^®^50, 0.1 mL/kg, Virbac, Carros, France). An approximately 10 mL blood sample was collected from each broiler via cardiac puncture, placed into serum separation tubes, centrifuged at 3,000 × *g* for 15 min at 4°C, and then stored at −80°C for subsequent biochemical and ELISA analyses. Following blood collection, the broilers were humanely euthanized by neck dislocation. Abdominal fat and liver samples from each broiler were collected and weighed. The relative weight of abdominal fat and liver was calculated using the formula: relative organ weight = organ weight (g)/BW (kg). Each liver was divided into four portions for different analyses: one portion was fixed in 10% neutral formalin solution for paraffin embedding and histological examination; one portion was stored at −80°C for subsequent molecular analysis; one portion was stored at −80°C for subsequent frozen sectioning; and the final portion was stored at −20°C for subsequent lipid content analysis.

### 2.3 Serum biochemical analysis and ELISA

The concentrations of total protein (TP), albumin (ALB), aspartate aminotransferase (AST), glucose (GLU), triglyceride (TG), total cholesterol (TC), high-density lipoprotein cholesterol (HDL-C) and low-density lipoprotein cholesterol (LDL-C) in broiler serum were measured using relevant commercial kits (Wuyue medical equipment co., ltd, Chongqing, China) via an automatic biochemical analyzer (Mindray BS-240, Shenzhen, China). The level of very low-density lipoprotein (VLDL) in broiler serum was determined using a double-antibody sandwich ELISA kit (Sinobestbio, Shanghai, China), and the absorbance value was read by using a microplate reader (ThermoFisher, Waltham, MA).

### 2.4 Soxhlet extraction method

The crude fat content of the liver was determined using the Soxhlet extraction method. After thorough drying and grinding, a 1 g liver sample (m_0_) was wrapped in paper and weighed (m_1_). The wrapped sample was then placed into a Soxhlet extractor and submerged in anhydrous ether (Chron Chemicals, Chengdu, China). The extraction process was conducted at 55°C for 6 h. After cooling, the paper package was dried and reweighed (m_2_). The crude fat content of the liver (%) was calculated as (m_1_-m_2_)/m_0_ × 100.

### 2.5 Paraffin and frozen sections

Hepatic lipid content was assessed by analyzing hematoxylin–eosin (HE) stained sections (Bioss, Beijing, China) and Oil Red O staining sections (Servicebio, Wuhan, China). Briefly, liver tissues were prepared into 3 μm-thick paraffin sections through a series of steps, including gradient ethanol dehydration (70, 90, 100, 100%), xylene clearing, paraffin infiltration and embedding, section spreading, HE staining, and neutral resin mounting. Concurrently, 8 μm-thick sections were prepared through gradient ethanol dehydration (70, 90, 100, 100%), optimal cutting temperature (OCT) embedding on dry ice at −20°C, sectioning with a cryostat (Leica, Hesse, Germany), Oil Red O staining, and mounting with glycerin gelatin sealing agent. Finally, all sections were examined under a microscope (Leica DM500, Leica Microsystems, Wetzlar, Germany). Finally, quantitative statistical analysis was performed on Oil Red O stained and HE stained liver sections using ImageJ software (version 5.0, BIO-RAD, Hercules, California). This involved measuring the gray values of positive areas stained for lipid droplets and areas of cellular vacuolar degeneration within liver sections, followed by calculating their proportional percentages relative to the total area’s gray value. The results were subsequently visualized through bar chart representation.

### 2.6 Western blotting

Total proteins were extracted from liver tissue and cells as previously described (23). Samples were lysed in radio-immunoprecipitation assay (RIPA) lysis buffer (ThermoFisher, Waltham, MA). Protein concentrations were determined by using a protein assay kit (Beyotime, Shanghai, China). Proteins were then separated on 10% SDS-polyacrylamide gels (Bioss, Beijing, China) and transferred onto polyvinylidene fluoride (PVDF) membranes (Solarbio, Beijing, China). The membranes were blocked with a 5% skim milk powder solution at room temperature for 2 h. Subsequently, membranes were incubated with primary antibodies at 4°C for 12 h. After incubation with corresponding secondary antibodies at RT for 1 h. The primary antibodies used in this study included: ADPN (bs-0471R; Bioss, Beijing, China), PPAR*α* (bs-3614R; Bioss, Beijing, China), TNF-α (bsm-33207 M; Bioss, Beijing, China), p-AMPK (bs-5551R; Bioss, Beijing, China), AMPK (bs-41337R; Bioss, Beijing, China), p-JNK1 (bs-17591R; Bioss, Beijing, China), JNK1 (bs-20760R; Bioss, Beijing, China), and GAPDH (bsm-33033 M; Bioss, Beijing, China) as an internal control. Secondary antibodies were HRP-labeled Goat Anti-Rabbit IgG (H + L) (A0208; Beyotime, Shanghai, China) or HRP-labeled Goat Anti-Mouse IgG (H + L) (A0216; Beyotime, Shanghai, China). Finally, the gray value of the bands was analyzed and counted by using ImageJ software. The gray value of the target protein band was normalized against the corresponding loading control band (GAPDH), and the resulting normalized values were utilized for graphical representation.

### 2.7 LMH cell culture and design

The Leghorn male hepatoma cell (LMH) line was purchased from iCell Bioscience Inc. (Shanghai, China) and cultured in LMH cell culture medium (iCell Bioscience Inc., Shanghai, China) at 37°C with 95% air and 5% CO_2_ in 0.1% gelatin-coated (Coolaber, Beijing, China) cell culture flasks. Cells were transferred to similarly treated cell culture plates at a density of 2 ~ 3 × 10^5^ cells/mL and divided into three groups: control group (CN, LMH cell culture medium); FE group (FE, 10% FE, v/v, Libangyingte, Xi’an, China) and FE + AdipoRon group (FE-AR, 10% FE and 4 μM AdipoRon, v/v) (23). Cells were incubated under these conditions for 24 h before collection for subsequent analysis. Three independent cell culture experiment (*n* = 3) was conducted, with two replicate cell wells for each cell experiment.

### 2.8 LMH cells viability, and steatosis

Different group LMH cells were cultured in 96-well plates for 24 h. Following the cell counting kit-8 (CCK-8) assay kit’s instructions (Biosharp, Hefei, China), the optical density (OD) values were measured using a microplate reader to determine the LMH cells viability. The lipid steatosis in LMH cell was then observed using an Oil Red O staining kit (Nanjing Jiancheng Bioengineering Institute, Nanjing, China).

### 2.9 Statistical analysis

The data were analyzed by using one-way analysis of variance (ANOVA) with the SPSS 25 software (IBM Co., Armonk, New York) to analyze the differences. Followed by the Tukey’s test for post-hoc multiple comparisons to assess normality. All data were presented as mean ± standard error of the mean (Mean ± SEM) by using GraphPad Prism 6.01 software (GraphPad Software, San Diego, California). *p* < 0.05 was considered statistically significant.

## 3 Results

### 3.1 Effects of AdipoRon on gross organ changes, hepatic function and lipid biochemical indices in CORT broilers

Compared with the CONT group, broilers in the CORT group exhibited a significant increase in liver weight, liver index, and serum levels of TP, ALB, TG, TC, HDL-C and LDL-C (*p* < 0.05, Table 2), and a decrease in plasma VLDL (*p* < 0.05). Meanwhile, compared with CORT broilers, CORT-AR group had a significant increase in plasma GLU concentration (*p* < 0.05), but no significant changes were observed in other indices (*p* > 0.05). Additionally, no significant differences in abdominal fat weight or abdominal fat percentage were detected among the three groups (*p* > 0.05).

**Table 2:** Effect of AdipoRon on BW, liver weight, abdominal weight, liver and lipid function indexes in serum of CORT broiler.

Item	CONT	CORT (4 mg/kg)	CORT + AdipoRon (0.2 mg/kg)	*p*
Average daily gain (g)	60.13 ± 16.65	28.77 ± 7.72	21.40 ± 4.72	0.144
Liver weight (g)	61.19 ± 4.81^b^	91.95 ± 5.32^a^	91.20 ± 10.34^a^	0.025
Liver index (%)	2.70 ± 0.11^b^	5.21 ± 0.31^a^	5.47 ± 0.50^a^	<0.001
Abdominal fat weight (g)	32.27 ± 7.12	37.13 ± 2.84	31.51 ± 1.64	0.307
Abdominal fat index (%)	1.31 ± 0.24	2.08 ± 0.10	1.93 ± 0.15	0.054
TP (g/L)	25.22 ± 1.21^b^	37.54 ± 1.34^a^	37.22 ± 1.21^a^	<0.001
ALB (g/L)	7.25 ± 0.31^b^	12.47 ± 0.70^a^	12.05 ± 0.52^a^	<0.001
AST (U/L)	232.98 ± 49.05^b^	311.27 ± 27.20^ab^	405.68 ± 45.70^a^	0.034
GLU (mmol/L)	13.78 ± 0.48^b^	19.36 ± 2.56^b^	35.56 ± 7.90^a^	0.037
TG (mmol/L)	1.16 ± 0.18^b^	3.44 ± 0.31^a^	4.27 ± 0.57^a^	<0.001
TC (mmol/L)	3.45 ± 0.09^b^	7.04 ± 0.15^a^	7.68 ± 0.43^a^	<0.001
HDL-C (mmol/L)	2.41 ± 0.07^b^	4.50 ± 0.15^a^	4.49 ± 0.25^a^	<0.001
LDL-C (mmol/L)	0.69 ± 0.05^b^	1.59 ± 0.18^a^	2.04 ± 0.25^a^	0.001
VLDL (mmol/L)	24.92 ± 0.99^a^	19.21 ± 1.73^b^	20.54 ± 1.00^ab^	0.019

The data represent mean ± SEM. Differences were determined by one-way ANOVA followed by Tukey’s test. The data within a row lacking a common superscript (a, b, c) indicates differences (*n* = 6, biological replicates, *p* < 0.05). CORT, corticosterone; AdipoRon, adiponectin receptor agonists; TP, total protein; ALB, albumin; AST, glutamic oxaloacetic transaminase; GLU, gluconate; TG, triglyceride; TC, total cholesterol; HDL-C, high density lipoprotein cholesterol; LDL-C, low density lipoprotein cholesterol; VLDL, very low-density lipoprotein.

### 3.2 AdipoRon decreases liver lipid accumulation in CORT broilers

**Figure 1:**
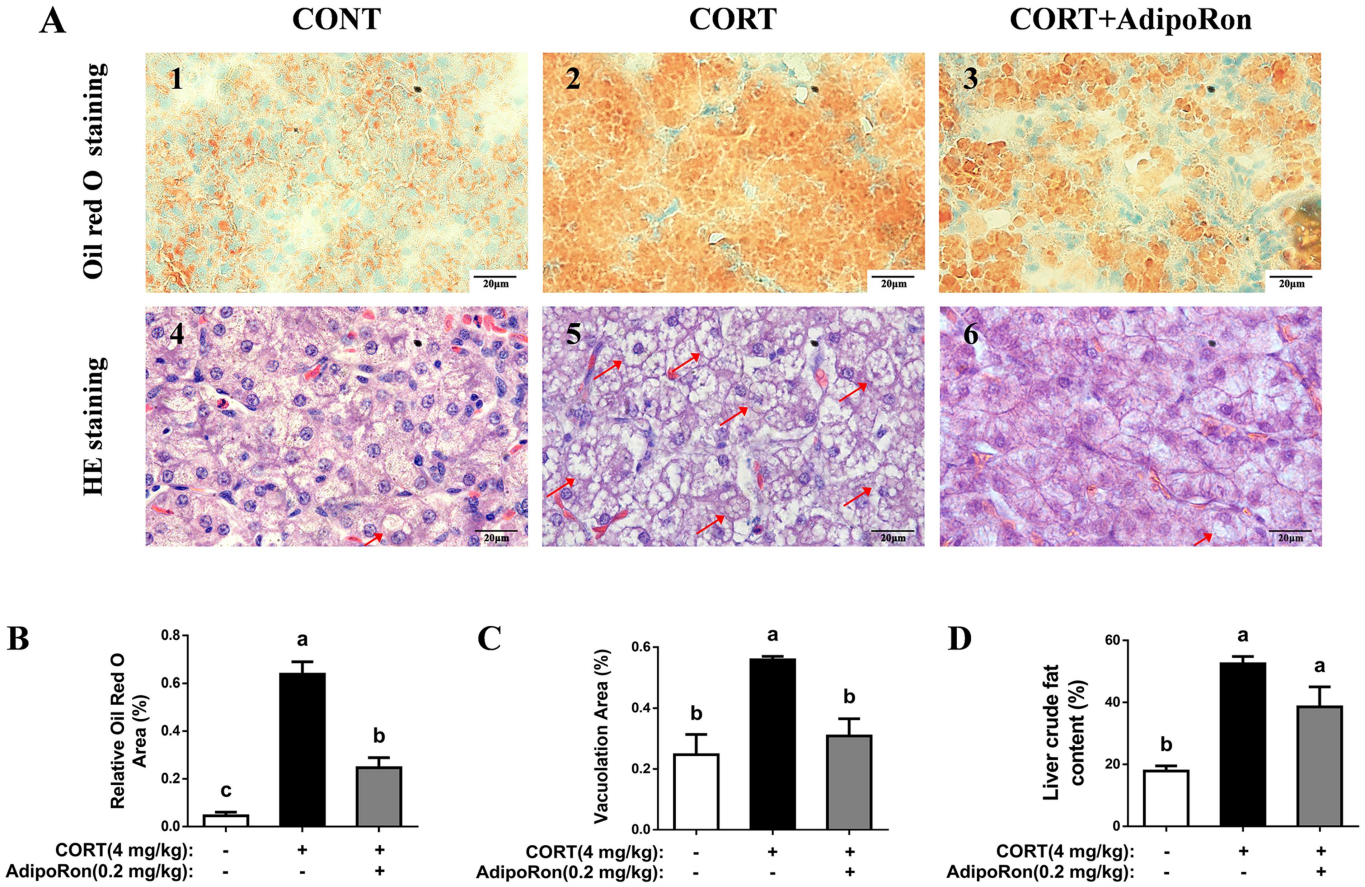
Effects of AdipoRon on lipid accumulation in the liver of CORT broilers. **(A1–3)** Liver oil red O staining in the liver of CORT broilers. **(A4–6)** Liver HE staining in the liver of CORT broilers, with arrows indicating areas of prominent vacuolization. Bar = 20 μm. **(B)** Relative Oil Red O area of CORT broilers. **(C)** Vacuolation area of CORT broilers. **(D)** Liver crude fat content of CORT broilers. The data represent mean ± SEM. Differences were determined by one-way ANOVA followed by Tukey’s test. The bars with different small letter (a, b, c) differ significantly between groups (*p* < 0.05, *n* = 6 per group, biological replicate). HE, hematoxylin and eosin.

Compared with the CONT broilers, Oil Red O staining of liver sections revealed increased lipid accumulation, indicated by more intense red staining, in the CORT broilers. In contrast, the CORT-AR group exhibited a noticeable reduction in red staining, suggesting decreased lipid deposition (*p* < 0.05, Figures 1A,B). HE stains revealed that the CORT group showed greater cytoplasmic vacuolation of hepatocytes, whereas the CORT-AR group exhibited a significant histological improvement (*p* < 0.05, Figures 1A,C). Additionally, liver crude fat content was significantly increased in the CORT group compared with the CONT group, while a significant reduction was observed in the CORT-AR group compared with the CORT group (*p* < 0.05, Figure 1D).

### 3.3 The effects of AdipoRon on proteins related to lipid catabolism and synthesis in liver of CORT broilers

**Figure 2:**
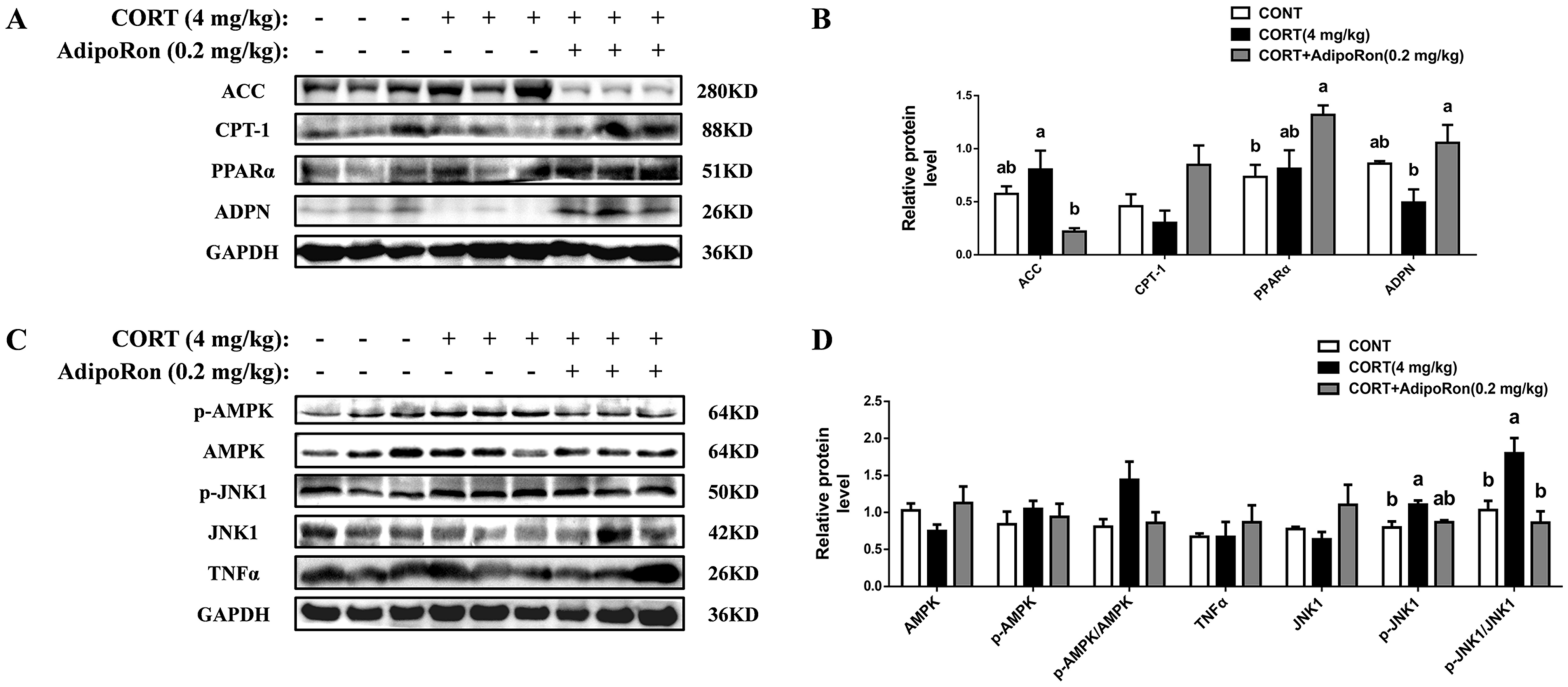
Effects of AdipoRon on lipid accumulation, AMPK/PPARα and JNK1 signaling pathway in the liver of CORT broilers. **(A)** Immunoblot of ACC, CPT-1, PPARα and ADPN protein level in the liver of CORT broiler. **(B)** The change of ACC, CPT-1, PPARα and ADPN protein expression in the liver of CORT broiler. **(C)** Immunoblot of p-AMPKα1, AMPKα1, p-JNK1, JNK1 and TNFα protein level in the liver of CORT broiler. **(D)** The change of p-AMPKα1, AMPKα1, p-JNK1, JNK1 and TNFα protein expression in the liver of CORT broiler. Grayscale values of each band were analyzed using ImageJ software. Normalization was performed by separately comparing the grayscale values of target protein bands with those of corresponding loading control bands (GAPDH), as well as the grayscale values of phosphorylated protein bands with those of total protein bands. The data represent mean ± SEM. Differences were determined by one-way ANOVA followed by Tukey’s test The bars with different small letter differ significantly between groups (*p* < 0.05, *n* = 6, biological replicates per group). ACC, Acetyl-CoA carboxylase 1; CPT-1, carnitine palmitoyl transferase-1; PPARα, peroxisome proliferators-activated receptor α; ADPN, adiponectin; AMPKα1, adenosine 5′-monophosphate (AMP)-activated protein kinase alpha 1; p-AMPKα1, phosphorylated adenosine 5′-monophosphate (AMP)-activated protein kinase alpha 1; JNK1, c-Jun N-terminal kinase 1; p-JNK1, phosphorylated c-Jun N-terminal kinase 1; TNF-α, Tumor Necrosis Factor-alpha.

Western blotting analysis showed that no significant differences in the protein expression levels of ACC, CPT-1, PPAR*α*, ADPN, TNF-α, JNK1, or the AMPK signaling pathway (*p* > 0.05, Figures 2A–D) were noted between the CONT and CORT groups, whereas the expression of p-JNK1 protein and the ratio of p-JNK1/JNK1 in the liver were significantly increased in CORT group (*p* < 0.05, Figures 2C,D). Meanwhile, compared with the CORT group, the expressions of ADPN proteins were significantly increased in the CORT-AR group (*p* < 0.05, Figures 2A,B), while the protein expressions of ACC and the ratio of p-JNK1/JNK1 were significantly decreased (*p* < 0.05, Figures 2A–D). However, no significant differences were detected on CPT-1, PPARα, the AMPK signaling pathway, or the inflammatory marker TNFα expression between the CORT and CORT-AT groups (*p* > 0.05).

### 3.4 The effects of AdipoRon on proteins related to lipid catabolism and synthesis in LMH cells

**Figure 3:**
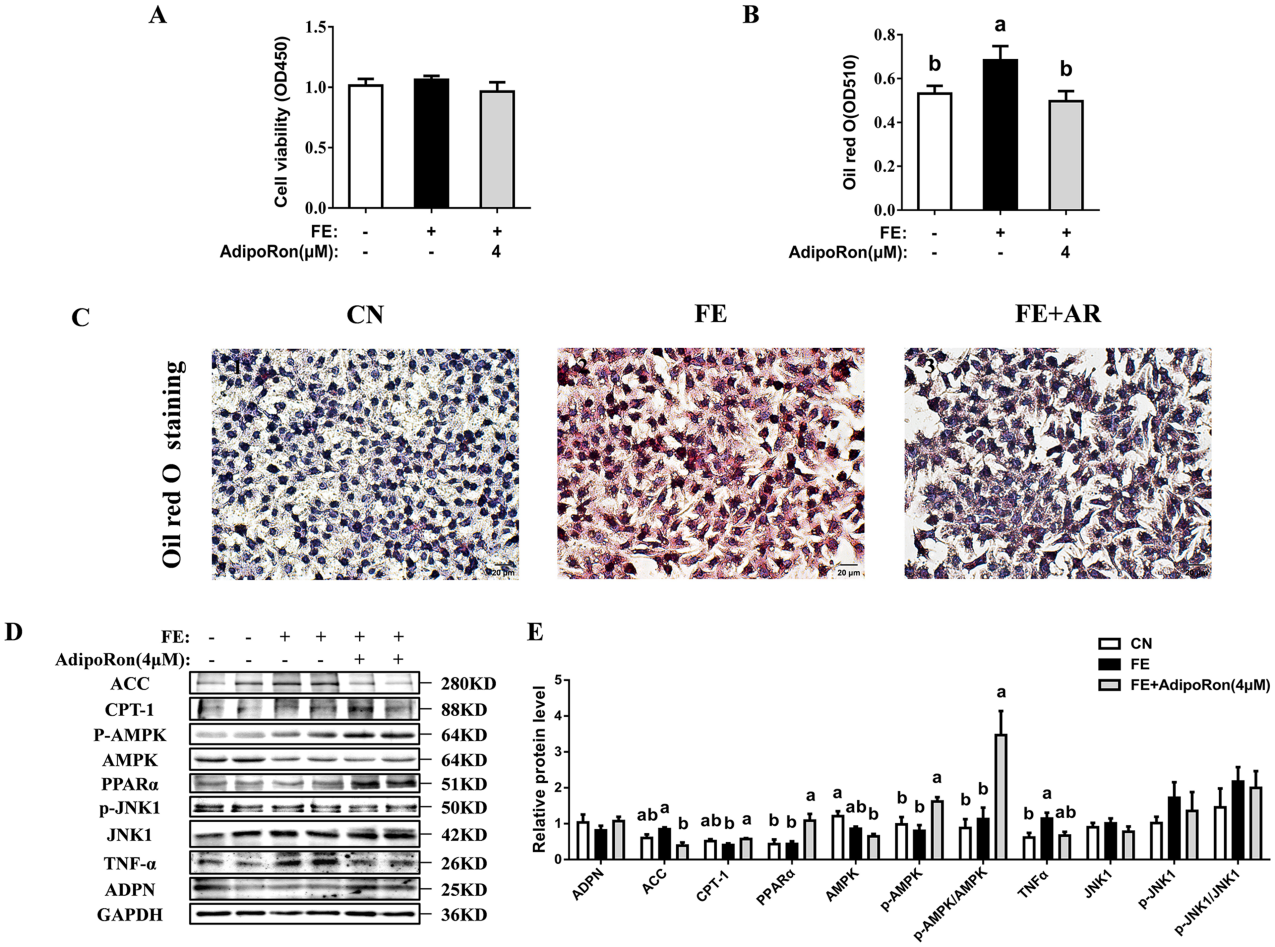
Effects of AdipoRon on viability, lipid accumulation, AMPK/PPARα and JNK1 signaling pathway in FE-induced LMH cells. **(A)** The cell viability. **(B)** The change of oil red O quantification in LMH cells. **(C1–3)** Liver oil red O staining in LMH cells. Bar = 20 μm. **(D)** Immunoblot of ACC, CPT-1, PPARα, ADPN, p-AMPKα1, AMPKα1 PPARα, p-JNK1, JNK1 and TNF-α protein level in LMH cells. **(E)** The change of ACC, CPT-1, PPARα, ADPN, p-AMPKα1, AMPKα1, PPARα, p-JNK1, JNK1 and TNF-α protein expression in LMH cells. Grayscale values of each band were analyzed using ImageJ software. Normalization was performed by separately comparing the grayscale values of target protein bands with those of corresponding loading control bands (GAPDH), as well as the grayscale values of phosphorylated protein bands with those of total protein bands. The data represent mean ± SEM. Differences were determined by one-way ANOVA followed by Tukey’s test The bars with different small letter (a, b, c) differ significantly between groups (*p* < 0.05, *n* = 6 per group, biological replicates). FE, fat emulsion; AdipoRon, adiponectin receptor agonists; ACC, Acetyl-CoA carboxylase 1; CPT-1, carnitine palmitoyl transferase-1; PPARα, peroxisome proliferators-activated receptor α; ADPN, adiponectin; TNF-α, tumor necrosis factor-alpha; AMPKα1, adenosine 5′-monophosphate (AMP)-activated protein kinase alpha 1; p-AMPKα1, phosphorylated adenosine 5′-monophosphate (AMP)-activated protein kinase alpha 1; JNK1, c-Jun N-terminal kinase 1; p-JNK1, phosphorylated c-Jun N-terminal kinase 1.

The CCK-8 assay showed that, compared to the CN cells, cell viability was significantly higher in the FE group (*p* < 0.05, Figure 3A) while the addition of AdipoRon did not result in a significant change in cell viability (*p* > 0.05). Oil Red O staining indicated that FE cells exhibited higher red staining intensity compared with CN cells, while in the FE-AR group, the red staining intensity was lower than that observed in the FE group (*p* < 0.05, Figures 3B,C). Western blotting analysis showed that compared with the CN cells, FE treatment was associated with higher TNF-*α* protein expression and p-AMPKα1/AMPKα1 ratio (*p* < 0.05, Figures 3D,E). In the FE-AR group, ACC expression was lower than that in the FE group (*p* < 0.05), and the expression level of TNF-α showed a downward trend but did not achieve statistical significance (*p* = 0.055). Additionally, CPT-1 and PPARα protein expression, and the ratio of p-AMPKα1/AMPKα1 were higher in the FE-AR group compared with the FE group (*p* < 0.05).

## 4 Discussion

Fatty liver has become a growing concern in the poultry industry. The demand for rapid growth and muscle accretion in broilers often necessitates the use of high-energy diets to meet metabolic needs. Unlike most other domestic animals, chickens synthesize most of their fat in the liver rather than in adipose tissue, making them more vulnerable to hepatic fat accumulation and, consequently, FLS (28). One of the pathological hallmarks of FLS is excessive lipid deposition within hepatocytes (29). To study the underlying mechanisms of FLS, 4.0 mg/kg CORT has been utilized to induce experimental models of fatty liver in chickens (30). Our results demonstrated that administration of CORT increased BW and liver index in broilers and led to enhanced lipid deposition, as evidenced by intensified Oil Red O staining and greater vacuolation observed via HE staining. Furthermore, CORT significantly increased serum levels of TP, ALB, TG, TC, LDL-C, and HDL-C, consistent with a hyperlipidemic state. Collectively, these findings indicated that CORT successively induced FLS in broilers, consistent with previous studies (26, 31).

AdipoRon is a synthetic ADPN receptor agonist that mimics the effects of endogenous ADPN (20). ADPN functions as an autocrine and paracrine regulator, playing a critical role in adipocyte metabolism and adipose tissue mass regulation (32, 33). In the present study, AdipoRon effectively prevented the increase in crude liver fat and hepatocyte lipid deposition induced by CORT in broilers. This aligns with the findings by Cao et al., who reported that AdipoRon reduced hepatic lipid deposition in geese FLS induced by a high-fat diet (HFD) (22). However, in our study, AdipoRon treatment did not reverse the CORT-induced increase in liver weight or liver index, suggesting that its effects may be specific to modulating lipid metabolism rather than reducing overall liver mass. Notably, no significant changes were observed in serum lipid parameters compared to the CORT group, which contrasts with earlier study in Huoyan geese where a 3-day treatment of AdipoRon reduced lipid content in both blood and liver tissues of high-fat diet-fed Huoyan Geese (22). Several factors may explain this discrepancy. First, differences in the FLS induction method could play a role. CORT-induced FLS may be more resistant to systemic lipid regulation by AdipoRon compared with diet-induced FLS. Second, treatment duration and timing are likely critical. In mice, a 10-day treatment markedly improved systemic lipid metabolism disrupted by high-fat diet, whereas a 20-day treatment reversed these beneficial effects (Wang et al., 2022). In our study, AdipoRon was administered for only 5 days, which may have been sufficient to reduce hepatic lipid accumulation but not long enough to significantly influence circulating lipid profiles. In addition, pre-treatment with AdipoRon (administered prior to the induction of liver injury) significantly lowered the serum markers of liver damage such as AST and ALT in mice (34, 35). Together, these findings suggest that while AdipoRon reliably reduces hepatic lipid deposition, its effects on systemic lipid metabolism may depend on the underlying etiology of FLS and the duration and timing of treatment. Further studies with larger sample sizes, varied treatment periods, and alternative induction models will be valuable to validate these findings and define the optimal conditions for AdipoRon efficacy in poultry. Additionally, the lack of an AdipoRon-only treatment group in healthy broilers limits interpretation of its effects under physiological conditions. Future investigations should include an AdipoRon monotherapy group under both physiological and pathological conditions to more clearly evaluate its direct effects and underlying mechanisms of action.

Research has shown that AdipoRon is able to activate receptors of ADPN, AdipoR1 and AdipoR2 (36). This activation subsequently triggers downstream signaling pathways, including AMPK and PPARα, ultimately modulating the expression of key lipid metabolism regulators such as ACC, a marker of fatty acid synthesis, and CPT1, a key enzyme involved in fatty acid oxidation (22). PPARα plays a crucial role in regulating fatty acid oxidation, lipid metabolism, and glucose homeostasis (37). The activation of PPARα reduces fat accumulation by enhancing hepatic fatty acid *β*-oxidation via expression of CPT1a and ACO (38, 39). AMPK is a central energy sensor activated under conditions of low cellular energy. This activation typically occurs through phosphorylation at a specific threonine residue on one of its subunits (40). Once activated, it promotes ATP-generating processes, such as fatty acid oxidation and autophagy, while inhibiting energy-consuming biosynthetic pathways, including gluconeogenesis, lipogenesis, and protein synthesis (41). In our study, AdipoRon upregulated hepatic PPARα and ADPN protein expression in the liver of CORT-treated broilers. *In vitro*, AdipoRon treatment significantly lowered intracellular lipid accumulation in FE-challenged LMH cells while upregulating CPT-1 and PPARα expression, suggesting enhanced lipid catabolism and reduced lipid deposition. In addition, AdipoRon increased the p-AMPKα1/AMPKα1 ratio and downregulated ACC expression in fat emulsified hepatocytes, indicating activation of AMPK and suppression of lipogenesis. Importantly, AdipoRon did not compromise hepatocyte viability in this model, suggesting that its lipid-lowering effects were not due to cytotoxicity. These findings align with previous studies showing that AdipoRon enhanced the phosphorylation of AMPK and ACC, and inhibited lipid accumulation in C3H10T1/2 cells and in mice model of acute liver injury by downregulating the expression of adipogenic transcription factors (42, 43). Moreover, AdipoRon has been reported to upregulate PPARα protein expression and downregulate ACC expression in FL83B mouse hepatocytes and in HFD-fed mice (21, 44). However, in our FLS broiler model, AdipoRon treatment did not significantly alter AMPKα1 or p-AMPKα1 protein expression levels. The observed discrepancies between *in vivo* and *in vitro* results may reflect the physiological complexity of avian model, where hormonal, metabolic, and immune interactions could modulate signaling cascades. For example, simultaneous activation of parallel pathways such as MAPK/ERK by AdipoRon has been reported in various cancer models, suggesting a potential for pathway cross-talk that may influence or obscure AMPK signaling outcomes *in vivo* (22, 45). Subsequent investigations should employ proteomics to identify avian-specific targets of AdipoRon and mitigate confounding effects from off-target interactions. Notably, CORT has been reported to antagonize AMPK activation (46). In CORT-induced stress model using PC12 cells, p-AMPK protein expression was also suppressed in a dose-dependent manner (47). Therefore, persistent high-dose CORT could have blunted AdipoRon’s ability to activate AMPK *in vivo*.

Inflammation is another key pathological feature of NAFLD and fatty liver hemorrhagic syndrome (FLHS). During the inflammatory process, the pro-inflammatory cytokine TNF-*α* is often overexpressed in response to various stimuli and contributes to the activation of multiple signaling pathways, including JNK (48). Activated JNK promotes the expression of pro-inflammatory genes through transcription factors such as c-Jun and ATF2, thereby amplifying the inflammatory response (49). Zhang et al. (50) demonstrated that the JNK inhibitor SP600125 suppressed the phosphorylation of JNK, reduced the levels of TNF-α and TG, and alleviated inflammation and lipid deposition in primary chicken embryonic hepatocytes. Similarly, AdipoRon has been shown to inhibit activation of TNF-α and JNK pathways in lipopolysaccharide (LPS)-stimulated human podocytes AB 8/13, as well as reduce the expression of TNFα and other inflammatory factors, alleviating inflammation in the kidneys of HFD-fed mice (51). Our *in vivo* findings revealed suppressed p-JNK1 levels in AdipoRon-treated broilers, suggesting suppression of JNK1 signaling pathway. Mechanistically, hepatic JNK suppresses PPAR*α* activity via the corepressors NCoR1 and NRIP1, leading to reduced fatty acid oxidation and increased lipid synthesis (52). Therefore, the observed upregulation of PPARα and reduction of hepatic lipid synthesis following AdipoRon treatment may be linked to inhibition of JNK1 signaling. Future studies should extend these findings by measuring additional lipid oxidation and synthesis-related markers, and by validating the mechanism with JNK-specific agonists and inhibitors to further define AdipoRon’s effects on JNK-mediated lipid metabolism. On the other hand, although AdipoRon exhibited a trend towards inhibition in the elevated expression of TNF-*α* induced by FE in LMH cells, this effect did not achieve statistical significance. This may suggest that AdipoRon has a certain inhibitory effect on TNF-α expression, further supporting its potential anti-inflammatory properties, although this effect may require further validation under conditions of larger sample sizes or using additional pro-inflammatory markers such as IL-1β and IL-6. In contrast, TNF-α protein expression *in vivo* did not significantly differ among the control, CORT, and CORT-AR groups. A potential explanation for this discrepancy is the dual role of GCs, which are known to induce hepatic steatosis while simultaneously exerting some anti-inflammatory effects (53). Although CORT at 4 mg/kg effectively induced hepatic lipid accumulation in our model as previously reported (26, 54, 55) – its anti-inflammatory properties may have masked the inflammatory response typically associated with FLS. Mei et al.’s (30) study showed that CORT-induced FLS broilers did not cause changes in the inflammatory response such as the hepatic expression of C-reactive protein (CRP), serum amyloid A (SAA), interleukin-1 (IL-1), interferon-*γ* (IFN-γ), interleukin-1β (IL-1β) and interleukin-6 (IL-6) and nuclear factor kappa-B (NF-κB) mRNA. Similarly, Chen et al.’s (56) experiment showed that CORT tended to reduce the content of TNF-*α* in the blood of broilers. Nevertheless, the results of *in vitro* experiments indicate that AdipoRon has the potential to alleviate the inflammatory response in hepatocytes. However, this result may need to be further verified using fatty liver models with a stronger inflammatory phenotype, such as FLS complicated by infection or other immune challenges. A limitation of this study is that we did not examine downstream AMPK targets such as SREBP-1c or FAS, which would have provided further mechanistic insight. Future work should include these markers and alternative FLS models to more comprehensively evaluate the anti-inflammatory role of AdipoRon in poultry.

In conclusion, AdipoRon may potentially possess the function of reducing hepatic lipid accumulation in CORT-induced FLS broilers by activating PPAR*α* and inhibiting lipid synthesis through downregulating ACC.

## Data Availability

The original contributions presented in the study are included in the article/supplementary material, further inquiries can be directed to the corresponding author/s.

## Glossary

FLS: fatty liver syndrome
GC: glucocorticoid
ADPN: Adiponectin
AdipoR1/2: ADPN receptors R1 and R2
AdipoRon: Adiponectin receptor agonist
FE: fat emulsion
CORT: corticosterone
LMH: Leghorn male hepatoma cells
ACC: Acetyl-CoA carboxylase 1
PPARα: peroxisome proliferator-activated receptor α
FAS: fatty acid synthetase
CPT-1: carnitine palmitoyl transferase 1
AMPKα1: adenosine 5′-monophosphate-activated protein kinase alpha 1
p-AMPKα1: Phosphorylated AMPK alpha 1
TNF-α: tumor necrosis factor-alpha
JNK1: c-Jun N-terminal kinase 1
p-JNK 1: phosphorylated JNK1
ACO: acetyl CoA oxidase
DMSO: dimethyl sulfoxide
PEG300: polyethylene glycol 300
ALB: albumin
AST: aspartate aminotransferase
TP: total protein
GLU: glucose
TG: triglyceride
TC: total cholesterol
HDL-C: high-density lipoprotein cholesterol
LDL-C: low-density lipoprotein cholesterol
VLDL: very low-density lipoprotein
OCT: optimal cutting temperature
RIPA: radio-immunoprecipitation assay
PVDF: polyvinylidene fluoride
PBS: phosphate-buffered saline
PBST: PBS with Tween-20
CCK-8: cell counting kit-8
ANOVA: one-way analysis of variance
LPS: lipopolysaccharide
CRP: cAMP receptor protein
SAA: serum amyloid A
IL-1: interleukin-1
IFN-γ: interferon-γ
IL-1β: interleukin-1β
IL-6: interleukin-6
NF-κB: nuclear factor kappa-B

## References

[1] Liu Y Wang Y Wang C Sun X Gao S Liu R. Alterations in hepatic transcriptome and cecum microbiota underlying potential ways to prevent early fatty liver in laying hens. Poult Sci. (2023) 102:102593. doi: 10.1016/j.psj.2023.102593, PMID: 36972673 PMC10066560

[2] Lv Y Ge C Wu L Hu Z Luo X Huang W. Hepatoprotective effects of magnolol in fatty liver hemorrhagic syndrome hens through shaping gut microbiota and tryptophan metabolic profile. J Anim Sci Biotechnol. (2024) 15:120. doi: 10.1186/s40104-024-01074-9, PMID: 39238062 PMC11378483

[3] Yue K Cao QQ Shaukat A Zhang C Huang SC. Insights into the evaluation, influential factors and improvement strategies for poultry meat quality: a review. NPJ Sci Food. (2024) 8:62. doi: 10.1038/s41538-024-00306-6, PMID: 39251637 PMC11385947

[4] Trott KA Giannitti F Rimoldi G Hill A Woods L Barr B. Fatty liver hemorrhagic syndrome in the backyard chicken: a retrospective histopathologic case series. Vet Pathol. (2014) 51:787–95. doi: 10.1177/0300985813503569, PMID: 24091813

[5] Lin CW Huang TW Peng YJ Lin YY Mersmann HJ Ding ST. A novel chicken model of fatty liver disease induced by high cholesterol and low choline diets. Poult Sci. (2021) 100:100869. doi: 10.1016/j.psj.2020.11.046, PMID: 33516481 PMC7936157

[6] Guo L Kuang J Zhuang Y Jiang J Shi Y Huang C. Serum metabolomic profiling to reveal potential biomarkers for the diagnosis of fatty liver Hemorrhagic syndrome in laying hens. Front Physiol. (2021) 12:590638. doi: 10.3389/fphys.2021.590638, PMID: 33633583 PMC7900428

[7] Tan X Liu R Xing S Zhang Y Li Q Zheng M. Genome-wide detection of key genes and epigenetic markers for chicken fatty liver. Int J Mol Sci. (2020) 21:1800. doi: 10.3390/ijms21051800, PMID: 32151087 PMC7084419

[8] Cai J Hu Q Lin H Zhao J Jiao H Wang X. Adiponectin/adiponectin receptors mRNA expression profiles in chickens and their response to feed restriction. Poult Sci. (2021) 100:101480. doi: 10.1016/j.psj.2021.101480, PMID: 34700095 PMC8554277

[9] Kang H Lee J. Adipose tissue macrophage heterogeneity in the single-cell genomics era. Mol Cells. (2024) 47:100031. doi: 10.1016/j.mocell.2024.100031, PMID: 38354858 PMC10960114

[10] Mishra G Townsend KL. Sensory nerve and neuropeptide diversity in adipose tissues. Mol Cells. (2024) 47:100030. doi: 10.1016/j.mocell.2024.100030, PMID: 38364960 PMC10960112

[11] Naing YT Sun L. The role of splicing factors in adipogenesis and thermogenesis. Mol Cells. (2023) 46:268–77. doi: 10.14348/molcells.2023.2195, PMID: 37170770 PMC10183792

[12] Chen CY Chen YJ Ding ST Lin YY. Expression profile of adiponectin and adiponectin receptors in high-fat diet feeding chickens. J Anim Physiol Anim Nutr (Berl). (2018) 102:1585–92. doi: 10.1111/jpn.12979, PMID: 30151936

[13] Ramachandran R Maddineni S Ocón-Grove O Hendricks G 3rd Vasilatos-Younken R Hadley JA. Expression of adiponectin and its receptors in avian species. Gen Comp Endocrinol. (2013) 190:88–95. doi: 10.1016/j.ygcen.2013.05.004, PMID: 23707376

[14] Rodríguez A Catalán V Becerril S Gil MJ Mugueta C Gómez-Ambrosi J. Impaired adiponectin-AMPK signalling in insulin-sensitive tissues of hypertensive rats. Life Sci. (2008) 83:540–9. doi: 10.1016/j.lfs.2008.07.022, PMID: 18761357

[15] Gamberi T Magherini F Modesti A Fiaschi T. Adiponectin Signaling pathways in liver diseases. Biomedicine. (2018) 6:52. doi: 10.3390/biomedicines6020052, PMID: 29735928 PMC6027295

[16] Ruan H Dong LQ. Adiponectin signaling and function in insulin target tissues. J Mol Cell Biol. (2016) 8:101–9. doi: 10.1093/jmcb/mjw014, PMID: 26993044 PMC4816150

[17] Dong Z Zhuang Q Ye X Ning M Wu S Lu L. Adiponectin inhibits NLRP3 inflammasome activation in nonalcoholic steatohepatitis via AMPK-JNK/ErK1/2-NFκB/ROS Signaling pathways. Front Med (Lausanne). (2020) 7:546445. doi: 10.3389/fmed.2020.546445, PMID: 33251225 PMC7674946

[18] Feng J Lu S Ou B Liu Q Dai J Ji C. The role of JNk Signaling pathway in obesity-driven insulin resistance. Diabetes Metab Syndr Obes. (2020) 13:1399–406. doi: 10.2147/dmso.S236127, PMID: 32425571 PMC7196768

[19] Barbalho SM Méndez-Sánchez N Fornari Laurindo L. AdipoRon and ADP355, adiponectin receptor agonists, in metabolic-associated fatty liver disease (MAFLD) and nonalcoholic steatohepatitis (NASH): a systematic review. Biochem Pharmacol. (2023) 218:115871. doi: 10.1016/j.bcp.2023.115871, PMID: 37866803

[20] Okada-Iwabu M Yamauchi T Iwabu M Honma T Hamagami K Matsuda K. A small-molecule AdipoR agonist for type 2 diabetes and short life in obesity. Nature. (2013) 503:493–9. doi: 10.1038/nature12656, PMID: 24172895

[21] Liu XH Pan JP Bauman WA Cardozo CP. AdipoRon prevents myostatin-induced upregulation of fatty acid synthesis and downregulation of insulin activity in a mouse hepatocyte line. Physiol Rep. (2019) 7:e14152. doi: 10.14814/phy2.14152, PMID: 31250564 PMC6597868

[22] Cao Z Ma B Cui C Zhao J Liu S Qiu Y. Protective effects of AdipoRon on the liver of Huoyan goose fed a high-fat diet. Poult Sci. (2022) 101:101708. doi: 10.1016/j.psj.2022.101708, PMID: 35150940 PMC8844248

[23] Tu WJ Zhang YH Wang XT Zhang M Jiang KY Jiang S. Osteocalcin activates lipophagy via the ADPN-AMPK/PPARα-mTOR signaling pathway in chicken embryonic hepatocyte. Poult Sci. (2024) 103:103293. doi: 10.1016/j.psj.2023.103293, PMID: 38070403 PMC10757024

[24] Shan X Xu X Wang L Lu Y Chen X Li F. Dietary curcumin supplementation attenuates hepatic damage and function abnormality in a chronic corticosterone-induced stress model in broilers. J Steroid Biochem Mol Biol. (2024) 243:106579. doi: 10.1016/j.jsbmb.2024.106579, PMID: 39032671

[25] Zhou Z Zhang A Liu X Yang Y Zhao R Jia Y. M(6)A-mediated PPARA translational suppression contributes to corticosterone-induced visceral fat deposition in chickens. Int J Mol Sci. (2022) 23:15761. doi: 10.3390/ijms232415761, PMID: 36555401 PMC9779672

[26] Hu Y Sun Q Liu J Jia Y Cai D Idriss AA. In ovo injection of betaine alleviates corticosterone-induced fatty liver in chickens through epigenetic modifications. Sci Rep. (2017) 7:40251. doi: 10.1038/srep40251, PMID: 28059170 PMC5216338

[27] Liu J Zhang K Zhao M Chen L Chen H Zhao Y. Dietary bile acids alleviate corticosterone-induced fatty liver and hepatic glucocorticoid receptor suppression in broiler chickens. J Anim Sci. (2024) 102:skae338. doi: 10.1093/jas/skae338, PMID: 39492782 PMC11604113

[28] Yin C Zhou C Shi Y Ge Y Gao X Wu C. Effects and potential mechanism of dietary vitamin C supplementation on hepatic lipid metabolism in growing laying hens under chronic heat stress. J Anim Sci. (2023) 101:skad308. doi: 10.1093/jas/skad308, PMID: 37843035 PMC10588821

[29] Yang F Ruan J Wang T Luo J Cao H Song Y. Improving effect of dietary soybean phospholipids supplement on hepatic and serum indexes relevant to fatty liver hemorrhagic syndrome in laying hens. Anim Sci J. (2017) 88:1860–9. doi: 10.1111/asj.12832, PMID: 28677164

[30] Mei W Hao Y Xie H Ni Y Zhao R. Hepatic inflammatory response to exogenous LPS challenge is exacerbated in broilers with fatty liver disease. Animals (Basel). (2020) 10:514. doi: 10.3390/ani10030514, PMID: 32204385 PMC7143745

[31] Wu L Liu X Zhang A Chen H Zhao R Jia Y. Chronic corticosterone exposure disrupts hepatic and intestinal bile acid metabolism in chicken. Front Vet Sci. (2023) 10:1147024. doi: 10.3389/fvets.2023.1147024, PMID: 37266385 PMC10229839

[32] Deng Y Scherer PE. Adipokines as novel biomarkers and regulators of the metabolic syndrome. Ann N Y Acad Sci. (2010) 1212:E1–e19. doi: 10.1111/j.1749-6632.2010.05875.x, PMID: 21276002 PMC3075414

[33] Khatun MA Sato S Konishi T. Obesity preventive function of novel edible mushroom, Basidiomycetes-X (Echigoshirayukidake): manipulations of insulin resistance and lipid metabolism. J Tradit Complement Med. (2020) 10:245–51. doi: 10.1016/j.jtcme.2020.03.004, PMID: 32670819 PMC7340980

[34] Sha M Gao Y Deng C Wan Y Zhuang Y Hu X. Therapeutic effects of AdipoRon on liver inflammation and fibrosis induced by CCl4 in mice. Int Immunopharmacol. (2020) 79:106157 31911372 10.1016/j.intimp.2019.106157

[35] Xiao W-Z Zhang L. Adiponectin receptor agonist AdipoRon relieves endotoxin-induced acute hepatitis in mice. Chin Med J. (2019) 132:2438–45. doi: 10.1097/CM9.0000000000000488, PMID: 31651516 PMC6831068

[36] Maddineni S Metzger S Ocón O Hendricks G 3rd Ramachandran R. Adiponectin gene is expressed in multiple tissues in the chicken: food deprivation influences adiponectin messenger ribonucleic acid expression. Endocrinology. (2005) 146:4250–6. doi: 10.1210/en.2005-0254, PMID: 15976057

[37] Li Y Pan Y Zhao X Wu S Li F Wang Y. Peroxisome proliferator-activated receptors: a key link between lipid metabolism and cancer progression. Clin Nutr. (2024) 43:332–45. doi: 10.1016/j.clnu.2023.12.005, PMID: 38142478

[38] Honda K Saneyasu T Sugimoto H Kurachi K Takagi S Kamisoyama H. Role of peroxisome proliferator-activated receptor alpha in the expression of hepatic fatty acid oxidation-related genes in chickens. Anim Sci J. (2016) 87:61–6. doi: 10.1111/asj.12392, PMID: 26031853

[39] Navidshad B Royan M. Peroxisome proliferator-activated receptor alpha (PPARα), a key regulator of lipid metabolism in avians. Crit Rev Eukaryot Gene Expr. (2016) 26:303–8. doi: 10.1615/CritRevEukaryotGeneExpr.2016016665, PMID: 27910744

[40] Hu X Liu L Song Z Sheikhahmadi A Wang Y Buyse J. Effects of feed deprivation on the AMPK signaling pathway in skeletal muscle of broiler chickens. Comp Biochem Physiol B: Biochem Mol Biol. (2016) 191:146–54. doi: 10.1016/j.cbpb.2015.10.007, PMID: 26497445

[41] Ashraf N Van Nostrand JL. Fine-tuning AMPK in physiology and disease using point-mutant mouse models. Dis Model Mech. (2024) 17:dmm050798. doi: 10.1242/dmm.050798, PMID: 39136185 PMC11340815

[42] Wang SJ Lu WY Liu KY. Adiponectin receptor agonist AdipoRon suppresses adipogenesis in C3H10T1/2 cells through the adenosine monophosphate-activated protein kinase signaling pathway. Mol Med Rep. (2017) 16:7163–9. doi: 10.3892/mmr.2017.7450, PMID: 28901521

[43] Wang Y Wan Y Ye G Wang P Xue X Wu G. Hepatoprotective effects of AdipoRon against d-galactosamine-induced liver injury in mice. Eur J Pharm Sci. (2016) 93:123–31. doi: 10.1016/j.ejps.2016.08.017, PMID: 27516150

[44] Okada-Iwabu M Iwabu M Ueki K Yamauchi T Kadowaki T. Perspective of small-molecule AdipoR agonist for type 2 diabetes and short life in obesity. Diabetes Metab J. (2015) 39:363–72. doi: 10.4093/dmj.2015.39.5.363, PMID: 26566493 PMC4641965

[45] Laurindo LF Sosin AF Lamas CB De Alvares Goulart R Dos Santos Haber JF Detregiachi CRP. Exploring the logic and conducting a comprehensive evaluation of AdipoRon-based adiponectin replacement therapy against hormone-related cancers-a systematic review. Naunyn Schmiedeberg's Arch Pharmacol. (2024) 397:2067–82. doi: 10.1007/s00210-023-02792-z, PMID: 37864589

[46] Yuan S-Y Liu J Zhou J Lu W Zhou H-Y Long L-H. AMPK mediates glucocorticoids stress-induced downregulation of the glucocorticoid receptor in cultured rat prefrontal cortical astrocytes. PLoS One. (2016) 11:e0159513. doi: 10.1371/journal.pone.0159513, PMID: 27513844 PMC4981361

[47] Ma RD Zhou GJ Qu M Yi JH Tang YL Yang XY. Corticosterone induces neurotoxicity in PC12 cells via disrupting autophagy flux mediated by AMPK/mTOR signaling. CNS Neurosci Ther. (2020) 26:167–76. doi: 10.1111/cns.13212, PMID: 31423743 PMC6978254

[48] Kim E Cho H Lee G Baek H Lee IY Choi EJ. TSG101 physically interacts with linear ubiquitin chain assembly complex (LUBAC) and upregulates the TNFα-induced NF-κB activation. Mol Cells. (2023) 46:430–40. doi: 10.14348/molcells.2023.0026, PMID: 37431163 PMC10336271

[49] Ahn S Kwon A Oh Y Rhee S Song WK. Microtubule acetylation-specific inhibitors induce cell death and mitotic arrest via JNK/AP-1 activation in triple-negative breast Cancer cells. Mol Cells. (2023) 46:387–98. doi: 10.14348/molcells.2023.2192, PMID: 36794420 PMC10258459

[50] Zhang M Tu WJ Zhang Q Wu XL Zou XY Jiang S. Osteocalcin reduces fat accumulation and inflammatory reaction by inhibiting ROS-JNK signal pathway in chicken embryonic hepatocytes. Poult Sci. (2022) 101:102026. doi: 10.1016/j.psj.2022.102026, PMID: 36174267 PMC9519800

[51] Lindfors S Polianskyte-Prause Z Bouslama R Lehtonen E Mannerla M Nisen H. Adiponectin receptor agonist AdipoRon ameliorates renal inflammation in diet-induced obese mice and endotoxin-treated human glomeruli ex vivo. Diabetologia. (2021) 64:1866–79. doi: 10.1007/s00125-021-05473-9, PMID: 33987714 PMC8245393

[52] Vernia S Cavanagh-Kyros J Garcia-Haro L Sabio G Barrett T Jung DY. The PPARα-FGF21 hormone axis contributes to metabolic regulation by the hepatic JNK signaling pathway. Cell Metab. (2014) 20:512–25. doi: 10.1016/j.cmet.2014.06.010, PMID: 25043817 PMC4156535

[53] Syed AP Greulich F Ansari SA Uhlenhaut NH. Anti-inflammatory glucocorticoid action: genomic insights and emerging concepts. Curr Opin Pharmacol. (2020) 53:35–44. doi: 10.1016/j.coph.2020.03.003, PMID: 32416533

[54] Feng Y Mei W Chen Q Chen X Ni Y Lei M. Probiotic supplementation alleviates corticosterone-induced fatty liver disease by regulating hepatic lipogenesis and increasing gut microbiota diversity in broilers. Microorganisms. (2025) 13:200. doi: 10.3390/microorganisms13010200, PMID: 39858968 PMC11767375

[55] Zhang R Sun J Wang Y Yu H Wang S Feng X. Ameliorative effect of phenolic compound-pterostilbene on corticosterone-induced hepatic lipid metabolic disorder in broilers. J Nutr Biochem. (2024) 137:109822. doi: 10.1016/j.jnutbio.2024.109822, PMID: 39645170

[56] Chen M He Y Jia Y Wu L Zhao R. Liver transcriptome response to avian pathogenic *Escherichia coli* infection in broilers with corticosterone treatment. Poult Sci. (2025) 104:105020. doi: 10.1016/j.psj.2025.105020, PMID: 40088534 PMC11937665

